# Exploring the Perspectives of Older Adults Living With HIV on Virtual Care: Qualitative Study

**DOI:** 10.2196/65730

**Published:** 2024-12-04

**Authors:** Kristina M Kokorelias, Dean Valentine, Erica M Dove, Paige Brown, Stuart McKinlay, Christine L Sheppard, Hardeep Singh, Andrew D Eaton, Laura Jamieson, Marina B Wasilewski, Alice Zhabokritsky, Ashley Flanagan, Reham Abdelhalim, Rahel Zewude, Rabea Parpia, Sharon Walmsley, Luxey Sirisegaram

**Affiliations:** 1 Department of Occupational Science and Occupational Therapy, Temerty Faculty of Medicine, University of Toronto, 500 University Avenue, Toronto, ON, M5G1X7, Canada, 1 416-586-4800 ext 4374; 2 Rehabilitation Sciences Institute, Temerty Faculty of Medicine, University of Toronto, Toronto, ON, Canada; 3 National Institute on Ageing, Toronto Metropolitan University, Toronto, ON, Canada; 4 Section of Geriatric Medicine, Department of Medicine, Sinai Health System and University Health Network, Toronto, ON, Canada; 5 KITE, Toronto Rehabilitation Institute, University Health Network, Toronto, ON, Canada; 6 Factor-Inwentash School of Social Work, University of Toronto, Toronto, ON, Canada; 7 Undergraduate Medical Education, Temerty Faculty of Medicine, University of Toronto, Toronto, ON, Canada; 8 Faculty of Social Work, Saskatoon Campus, University of Regina, Saskatoon, SK, Canada; 9 Ontario Federation of Indigenous Friendship Centres, Toronto, ON, Canada; 10 St. John’s Rehab Research Program, Sunnybrook Health Sciences Centre, Toronto, ON, Canada; 11 Infectious Diseases, Department of Medicine, University Health Network, Toronto, ON, Canada; 12 Division of Infectious Diseases, Department of Medicine, University of Toronto, Toronto, ON, Canada; 13 CIHR Canadian HIV Trials Network, Vancouver, BC, Canada; 14 Burlington Ontario Health Team, Joseph Brant Memorial Hospital, Burlington, ON, Canada; 15 St. Michael's Hospital, Toronto, ON, Canada

**Keywords:** HIV, human immunodeficiency virus, perspective, telemedicine, telehealth, virtual care, virtual health, virtual medicine, qualitative, gerontology, geriatrics, older adult, older people, aging

## Abstract

**Background:**

As the population of individuals with HIV ages rapidly due to advancements in antiretroviral therapy, virtual care has become an increasingly vital component in managing their complex health needs. However, little is known about perceptions of virtual care among older adults living with HIV.

**Objective:**

This study aimed to understand the perceptions of older adults living with HIV regarding virtual care.

**Methods:**

Using an interpretive, qualitative, descriptive methodology, semistructured interviews were conducted with 14 diverse older adults living with HIV. The participants lived in Ontario, Canada, self-identified as HIV-positive, and were aged 50 years or older. Efforts were made to recruit individuals with varying experience with virtual health care. Reflexive thematic analysis was conducted with the interview transcripts to identify prevalent themes.

**Results:**

The identified themes included (1) the importance of relationships in virtual care for older adults living with HIV; (2) privacy and confidentiality in virtual care; and (3) challenges and solutions related to access and technological barriers in virtual care. These themes highlight the perceptions of diverse older adults living with HIV concerning virtual care, emphasizing the fundamental role of trust, privacy, and technology access.

**Conclusions:**

By embracing the unique perspectives and experiences of this population, we can work toward building more inclusive and responsive health care systems that meet the needs of all individuals, regardless of age, HIV status, or other intersecting identities.

## Introduction

The increased uptake in virtual care in response to the COVID-19 pandemic represents a shift in health care delivery worldwide [1-4]. This expansion of virtual care necessitated numerous assessments of its efficacy and suitability for patients with different disease states and demographics [5,6]. Among these studies, the emerging and concerning trend of older individuals lacking access to health care, including timely care, became notable [7], presenting a potential added risk of morbidity and mortality [7]. In contrast, the use of virtual care has continued to be advocated for following the pandemic to provide options and to increase the availability and accessibility of health care services for older adults [7,8]. We define virtual care as “the interaction between patients and/or members of their circle of care, occurring remotely, using any forms of communication or information technologies with the aim of facilitating and maximizing the quality and effectiveness of patient care,” in accordance to the Canadian Women’s College Hospital Institute for Health System Solutions and Virtual Care [9]. Virtual care can encompass various modalities, including teleconsultation, remote monitoring, and mobile health applications, among others [10,11].

Older adults represent a patient demographic with distinct health care needs and challenges who might benefit from virtual care [12]. As individuals age, they often experience age-related health conditions, chronic diseases, and functional limitations that necessitate frequent interactions with the health care system [13,14]. Moreover, older adults may face logistical obstacles, such as transportation challenges, lack of time, mobility limitations, and caregiver responsibilities, which can impede their access to in-person health care services [15-18]. Virtual care interventions tailored to older adults can offer numerous advantages, including improved access to care [19-21], enhanced convenience for homebound older adults [22] and those who live in rural and remote areas [23,24], cost-effectiveness [22], and the potential for early detection and intervention of clinically adverse events [25]. Thus, virtual care for specialized populations is valuable, as it increases access to specialists who might otherwise be inaccessible.

Older adults living with HIV are accessing virtual care services more commonly [26,27]. As the population of individuals with HIV ages, their complex health needs require specialized management [28]. The intersection of HIV and aging poses unique health care challenges [29-32], and as this population grows rapidly due to advancements in antiretroviral therapy, virtual care has become an increasingly vital component in managing their complex health needs [32]. However, little is known about the experiences and perceptions of older adults living with HIV regarding virtual care [26,33]. Addressing this knowledge gap may inform improvements to virtual care delivery by highlighting the unique health care needs of this population [26].

The goal of this study was to understand the perceptions of diverse older adults living with HIV regarding virtual care. Specifically, we aimed to explore the perceptions of diverse older adults living with HIV on (1) how virtual care supported age-related conditions experienced by older adults living with HIV; (2) perceived obstacles, pathways, and needs encountered in virtual care; and (3) recommendations for improving virtual care to support diverse older adults living with HIV.

## Methods

### Ethical Considerations

The study received ethics approval from the Mount Sinai Hospital Research Ethics Board (REB: 23-0106E).

### Methodology

We used an interpretive qualitative descriptive methodology for this study [34]. This methodology was selected as it is geared toward generating knowledge applicable to clinical practice, which aligns with our objectives [34].

This work was part of a broader research program[35] that involved a diverse advisory team of 10 individuals working within nonprofit community-based organizations, clinical settings, with lived and living experience, and working within policy, and research sectors. As such, an integrated knowledge translation approach was applied throughout this study [34]. This collaborative effort aimed to generate relevant and practical knowledge aligned with our study objectives. The advisory committee played a key role in identifying participants, analyzing data, reviewing the manuscript, and disseminating findings. Our team includes academic researchers and clinicians from geriatric, social work, and infectious disease programs, as well as partners from community organizations, lived experience of HIV, and policy sectors. Some individuals informed this project through their dual roles as researchers and lived experience representatives.

### Participants

The participants were not known to our research team in any personal, professional, or academic capacity before the study. All interactions with participants were strictly limited to the context of the research project. To be eligible, individuals had to self-identify as HIV-positive, be 50 years of age or older, and reside in Ontario. This geographic specificity facilitated a targeted examination of health care resources available to older persons living with HIV in this province.

### Recruitment

Our advisory committee’s websites and social media platforms were used to promote the study. We also recruited through HIV clinics, geriatric clinics, and other health care facilities catering to older individuals living with HIV, to engage potential participants already involved in health care services in Ontario. Representation was purposively sought across various areas to ensure diversity in perspectives [36], including sex and gender, age, ethnicity and race, socioeconomic status, prior usage of virtual-geriatric care, geographical location (rural vs urban), non-English first language, and level of educational attainment. This involved targeted recruitment from community-based organizations, including churches, mosques, temples, shelters, community centers, senior groups, health centers, libraries, and senior living buildings [37]. At these community-based locations, organizational staff posted flyers and circulated newsletters regarding the purpose of the study. Interested participants reached out to the research team via email or telephone. After the principal investigator or the research coordinator contacted interested persons to determine eligibility and to confirm interest via voluntary verbal consent, the participants were provided a written consent form and scheduled a time and location for the interview. The participants were given the option of an in-person, Zoom for Healthcare, or telephone interview. The participants were also offered the opportunity to bring a support person to the interview.

### Data Collection

Individual interviews (n=14) were conducted by a trained research assistant (n=10) or a peer researcher with lived experience (n=4) to enhance participants’ comfort in expressing their candid thoughts and opinions about virtual care. Interviews were guided using a semistructured interview guide that was developed by the research team in consultation with an advisory committee and took place between November 2023 and April 2024.

The semistructured interview guide (Table S1 in Multimedia Appendix 1) explored participants’ experiences with virtual care, including obstacles, pathways, and needs encountered in virtual care. Interview questions also examined their reasons for choosing or avoiding virtual care, the types of health care providers and conditions they consulted in their virtual appointments, and their overall care experiences during and after the COVID-19 pandemic when much care shifted to virtual formats.

The participants also had the option of completing an optional demographic form, either before, during, or after the interview.

Recruitment continued until data saturation was achieved [38]. Data saturation was determined through an ongoing review of the interviews by the research team to ensure no new data themes emerged [38]. The participants received a CAD $25 (US$ 17.80) gift card honorarium as a token of appreciation for their time. All interviews were recorded, professionally transcribed, and reviewed for accuracy by a research assistant.

### Data Analysis

Transcripts were analyzed and organized using NVivo 12 software [39]. The reflexive thematic analysis process outlined by Braun and Clarke was followed [40]. The research assistants and principal investigator read each transcript thoroughly, coded the data, and collaborated regularly with the co-investigators to develop and fine-tune the coding scheme. This involved recursive coding and theme development, including immersion in the data, review of relevant literature, and deep reflection [40]. Quality and reporting were guided by checklists provided by Braun and Clarke [41], including Braun and Clarke’s 15 questions for evaluating thematic analysis papers for publication (see Table S2 in Multimedia Appendix 1 [42,43]), and the Standards for Reporting Qualitative Research [44].

The sociodemographic characteristics of the participants were summarized using descriptive statistics.

### Rigor

To ensure methodological rigor, several strategies were used. These included building rapport with participants, seeking feedback on the interview guide, presenting direct quotes, engaging in team discussions to interpret data, and maintaining reflective notes throughout the analysis process. In addition, a collaborative and inclusive approach was maintained with participants, prioritizing open discussions and considering practical constraints such as scheduling, arranging interviews through a modality convenient to the participant, and the duration of interviews. This approach facilitated authentic exchanges, contributing to enhanced data quality.

## Results

### Overview

A total of 14 participants participated in this study. Of this, 4 identified as women and 10 as men. The participants were 63 years old on average (SD 10). Overall, 9 participants resided in urban settings, 2 in suburban settings, and 3 in rural settings. In total, 9 participants identified as low income (CAD $0-$29,999). Further details on participants are given in Table 1. Additional details are provided in Table S3 in Multimedia Appendix 1.

**Table 1.** Participant demographics.

Demographic characteristics	Participants (N=14), n (%)
**Age group (years)**	
50‐54	4 (29)
55‐59	4 (29)
60‐64	0 (0)
64‐69	3 (21)
70‐74	0 (0)
75‐79	1 (7)
80+	2 (14)
**Gender**	
Men	10 (71)
Women	4 (29)
Nonbinary	0 (0)
Two-spirit	0 (0)
**English as first language**	
Yes	8 (57)
No	6 (43)
**Access to a computer**	
Yes	9 (64)
No	1 (7)
No response	4 (29)
**Access to a smartphone**	
Yes	9 (64)
No	1 (7)
No response	4 (29)
**Access to internet connectivity**	
Yes	9 (64)
No	1 (7)
No response	4 (29)
**Require assistance with internet use**	
Yes	3 (21)
No	7 (50)
Prefer not to say	4 (29)
No response	0 (0)

### Theme 1: Importance of Relationships in Virtual Care for Older Adults Living With HIV

Privacy and data security emerged as key factors influencing the acceptance of virtual geriatric care, with some participants appreciating the privacy of virtual consultations while others preferred in-person visits to ensure confidentiality. Access to technology and internet connectivity were identified as significant obstacles, and participants noted the need for technology training and mutual understanding between patients and health care providers. Stigma associated with registering for age-related virtual care services was also a concern, with suggestions to integrate these services into existing health care frameworks to mitigate this issue. The findings are highlighted using participant quotes cited by the study participant ID, gender of the participant, and age.

All participants described experiencing unique health care challenges that intersect with both aging (eg, memory loss and frailty) and their HIV status (eg, stigma and risk of infection), making supportive and trustworthy relationships with health care providers crucial. The participants noted that the same health care providers should be accessible through virtual care to build effective communication, trust, and a sense of security. The participants who described having strong, positive relationships with their health care providers resulted in them being more likely to engage in open communication about their health concerns and feel supported in managing their condition(s). For example, one participant shared how trust in her relationship with her family physician allowed her to be tested for HIV:

*I could finally be honest of what was happening in all areas of my life and trusted [family doctor] enough to finally go and get tested anonymously*

[Participant 09, woman, 70 years]

On the other hand, when participants lacked a trusting relationship with their health care provider, they seldom adhered to their treatment or appointment schedule. One participant shared:

*It’s hard to want to go to the doctor when you feel like they are judging. The stories and stuff I have been told by doctors. Why would I ever go back and show up? Maybe I should put my pride aside, but it’s enough to make you rather be sick*

[Participant 11, male, 52 years]

The participants recognized feelings of discomfort during their interactions with new health care providers. One participant shared that he disliked having to “break in new doctors*,*” when discussing his experiences living with HIV (Participant 02, Man, 57 y old). The participants, therefore, emphasized that one of the critical aspects of building strong relationships in virtual health care is the consistency of health care providers. In addition to clinical discussions, the participants emphasized the importance of exploring social and emotional needs with their virtual health care provider. However, the participants who had experience with virtual care of any kind before the interview noted that the physical distance and lack of face-to-face interaction in virtual care made them feel disconnected from their care team, and sometimes challenged the trust that they had in their health care provider, questioning whether the health care provider was truly engaged in their care. One participant shared, “why would I just go and disclose all this to someone I never met and get their judgement” (participant 02, man, 57 years), highlighting the importance of consistent health care providers for discussing personal and sensitive health issues.

The participants noted that if they received care from a consistent health care provider, whether a physician or nurse, they would be willing to access other virtual care services (eg, from a geriatrician or therapist). Adding additional services and health care providers was often accompanied by discussions about who should be involved in a health care team. The integration of various health care providers into a patient’s care team emerged throughout the interviews, as the participants noted that they had some trusted health care professionals that they would continue to interact with in-person (eg, pharmacists), even if they saw the same or new health care providers virtually. The participants noted that other members of the care team need to be managed thoughtfully to support trusting relationships between patients and their health care providers.

*You can tell when a doctor really cares about me. Like when they just know me and actually remember things. So if I had a doctor who I knew actually cared, I’d be more open to using virtual services because I know they would tell me if I needed to come in. They care enough to be honest, not whatever is easiest for them. But like would I go to everyone virtually? Probably not.*

[Participant 06, female, 57 years]

### Theme 2: Privacy and Confidentiality in Virtual Care

Privacy emerged as a key theme that influenced participants’ acceptance of and preference for virtual care. Some participants noted that they would prefer virtual care, particularly phone consultations, over in-person care, as they would not have to see the care provider and thus, privacy and some degree of anonymity could be maintained. For example, one participant said:

*sometimes, I worry about them knowing me. So if I could just call someone with a question, maybe I’d appreciate it.*

[Participant 09, woman, 70 years]

These participants noted that they appreciated the enhanced privacy that virtual care could provide, such as the ability to discuss sensitive issues like menopause and sexual health, from the comfort and security of their own homes, without the risk of being overheard or recognized in a clinical setting. On the other hand, other participants, particularly those who spoke about living in close proximity with others (eg, partner or family members), noted that they preferred in-person appointments to ensure that their privacy and confidentiality were maintained from others in their lives. One participant shared:

*Even if you live with a partner you trust, there are things you want to say alone*

[Participant 01, man, 55 years]

In relation, some participants noted distrust in virtual care platforms as they were skeptical that these platforms could keep their information secure and private. These participants often shared that they heard from friends or family that companies sometimes sold patient health data. For others, despite being aware of the confidentiality and security measures in place for virtual care across Canada, the participants still harbored concerns that they could not ascertain what a provider would discuss with other health care providers.

#### Subtheme: Stigma of Registration

A few participants expressed that actively registering for a new virtual geriatric care service could heighten their sensitivity to stigma, as it marked them as individuals with an age-related issue, even if they did not see themselves as older. One participant noted:

*Bad enough they call the doctors infectious doctors, and now I have to see an old person doctor*

[Participant 10, man, 79 years]

To mitigate this, the participants suggested integrating the virtual clinic seamlessly with other primary health care services that they were already receiving. They proposed that all individuals living with HIV should be able to register for a virtual clinic, alongside other existing health care services, allowing them to be able to speak to someone about age-related concerns in a private manner.

### Theme 3: Access and Technological Barriers in Virtual Care: Challenges and Solutions

Almost all the participants reported that the COVID-19 pandemic had discouraged them from seeking or attending in-person health care, with less than half of the participants noting that they used virtual care. Despite this, most participants noted that they could access a telephone or computer to access virtual care, even if they had to share the technology with other individuals. For many participants, virtual care allowed them to reduce the time taken off from work to attend appointments and associated costs of appointments, such as gas and parking costs. Other participants, expressed that not having a strong internet connection or personal devices presented an obstacle to virtual care. The participants, without the appropriate technology to access virtual care, noted concerns about accessing a secure location to connect to virtual appointments. These participants also spoke to challenges associated with virtual care due to poor internet connectivity, audio problems, and outdated technology, particularly affecting those in rural areas or without access to high-speed internet. One participant explained:

*People need to think on a spectrum. Sometimes I may have Wi-Fi, sometimes I may not, sometimes I can’t afford my phone bill and that will be turned off. We need to consider something that can always be ther*e.

[Participant 14, man, 50 years]

#### Subtheme: Technology Training That Goes Both Ways

The participants expressed mixed feelings about the use of technology to access geriatric care. Some highlighted difficulties due to limited technological literacy and hoped health care providers could help them navigate virtual care and electronic health information. However, others were concerned that health care providers might assume they lacked technological skills based solely on their age or HIV status. They also expressed a need for guidance on effectively communicating relevant information to health care providers in virtual settings, especially without nearby support or written instructions. One participant said:

*My English isn’t great so I don’t even know how to ask for help. Sometimes in person I can write it down, but online, I don’t know*.

[Participant 04, male, 82 years]

Despite these concerns, the participants acknowledged that virtual care could play a role in educating health care providers about HIV. They believed that while virtual care might help facilitate this education, sharing personal experiences and knowledge in person was often more effective due to fewer distractions and a more direct communication channel. One participant emphasized this point, stating:

*They [physicians] can’t know everything, even if they read it in a textbook. They need to listen to people like us who are aging and who have HIV and sometimes a lot more*

[Participant 03, woman, 74 years]

This reflects a preference for in-person interactions when discussing complex, lived experiences, despite recognizing the potential benefits of virtual care.

## Discussion

### Principal Findings

The study examined the perceptions of diverse older adults living with HIV regarding virtual care, focusing on supporting age-related conditions and identifying obstacles and pathways. Key findings highlighted the importance of consistent and trustworthy relationships with health care providers, emphasizing the need for continuity in virtual care to build rapport. Privacy was a significant concern, with preferences for virtual or in-person care varying based on individual privacy needs and concerns about data security. Access to technology was another critical issue, with obstacles including poor connectivity, audio problems, and outdated devices, particularly affecting rural participants. The participants also highlighted the need for technological assistance and the opportunity to educate geriatric care providers about the intersection of aging and HIV. The study emphasizes that older adults living with HIV can offer valuable insights to enhance virtual care, helping to overcome obstacles such as distance, mobility, and transportation. Our themes, while addressing important aspects of virtual care, highlight that many issues transcend age demographics and are more specifically related to the virtual aspect of care itself rather than being uniquely tied to geriatric care.

Many of the themes uncovered in this study resonate with findings from prior research conducted among the general older adult population. Other studies with older adults have noted that the potential advantages reported by participants included enhanced convenience, and the ability to conduct consultations within the familiar setting of patients’ homes, supporting their comfort [45-47]. Similarly, studies noted that older adults have privacy concerns about the use of their health data [46,48,49]. Moreover, some older adults noted challenges due to a lack of technology and/or technological literacy [24,28]. While our study aligns with previous research on the general older adult population, it offers unique insights by focusing specifically on older adults living with HIV. As such, their perspectives on the importance of privacy and trust-building with health care providers during virtual care may differ from those of the general older adult population. Therefore, health care providers offering virtual care to this population must prioritize strategies to ensure the privacy and confidentiality of patient information, thereby fostering a sense of trust and confidence among older adults living with HIV. In practice, health care organizations offering virtual care should implement policies and protocols designed to safeguard patient privacy and promote trust-building between health care providers and patients, such as allowing additional time for visits and follow-up, and communicating with older adults where the provider is situated during calls [50]. These efforts strive to ensure that virtual care delivery is respectful, nonjudgmental, and tailored to the unique circumstances of each patient.

The study participants expressed a strong desire for consistency in their health care providers, underscoring the importance of trust and familiarity in managing their health conditions. This finding aligns with existing literature, which highlights that continuity of care is crucial for building patient-provider relationships, improving patient satisfaction, and enhancing health outcomes, especially for older adults with complex health needs [51-53]. However, given the scarcity of geriatricians [54,55], relying solely on geriatricians for consistent care is impractical. Older adults living with HIV can benefit from receiving care from specialized geriatric interprofessional teams [55], but the success of such care hinges on the establishment of trust and rapport between patients and health care providers. As a viable alternative, a system of “soft handovers” can be implemented. Soft handovers involve a thorough and empathetic transition process between health care providers, ensuring the new provider is well-informed about the patient’s history, preferences, and needs [56]. This approach can minimize disruption and maintain continuity of care, addressing the gap caused by the limited number of geriatric specialists. Implementing soft handovers can ensure that older adults living with HIV receive consistent and comprehensive care, despite the limited availability of specialized geriatricians. However, prior to the handoff, fostering trusting relationships between patients and members of the geriatric interprofessional team, including geriatricians, nurses, social workers, and other specialists, is essential. To build trust, geriatric interprofessional teams must prioritize patient-centered care, empathy, and cultural sensitivity [57,58]. This involves actively listening to patients’ concerns, respecting their autonomy, and involving them in decision-making regarding their care [59]. In addition, health care providers should be knowledgeable about the unique needs and experiences of older adults living with HIV, including the physical, psychological, and social dimensions of aging with a chronic illness [60]. By demonstrating competence and understanding in addressing these needs, health care providers can establish credibility and foster trust with their patients in a virtual space.

Numerous studies document how stigma associated with HIV can deter individuals from seeking care or disclosing their condition, leading to disparities in health care utilization and outcomes [61]. Moreover, research on age-related stigma highlights how societal perceptions of aging can influence individuals’ self-perception and willingness to engage with services tailored for older adults [62,63]. Strategies proposed by the participants in this study, such as integrating virtual clinics with existing health care services to reduce the visibility of age-related concerns, align with recommendations from previous studies in the realm of mental health aimed at destigmatizing health care access [64]. By incorporating virtual care into comprehensive health care delivery models, health care providers can create inclusive environments where individuals feel comfortable addressing their health needs without fear of judgment or discrimination. However, it is important to recognize that challenges to access may persist, particularly for the most marginalized populations. Individuals facing intersecting forms of stigma and discrimination, such as older adults living with HIV, may still encounter challenges in accessing virtual care services due to systemic inequalities, digital divides, and social determinants of health [65]. Future research should prioritize understanding efforts to address stigma and discrimination within health care settings to be integrated into virtual care initiatives, ensuring that all individuals, regardless of their background or health status, feel valued and respected in their interactions with health care providers. Collaborative partnerships between researchers, health care providers, policymakers, and community organizations are essential for identifying and codesigning solutions that address these obstacles effectively, ultimately advancing equity and accessibility in virtual care delivery.

### Limitations

Despite the valuable insights gained from this study, several limitations should be acknowledged. First, the sample consisted of older adults living with HIV in Ontario, which may limit the transferability of the findings to other geographic locations or populations with different health care systems. Another limitation is that all participants had access to technology for virtual care. In addition, most were younger than 60 years, and the majority had been living in Canada for over 10 years. These factors may not be representative of the older adult living with HIV population. In addition, the recruitment strategy primarily relied on community-based organizations and health care facilities, potentially introducing selection bias toward individuals already engaged in health care services. Despite challenges, such as participant availability and interest, efforts were made to encompass diverse perspectives in this study, although our sample may limit the transferability of the findings to other contexts. Furthermore, the study did not explore the perspectives of health care providers or other stakeholders involved in the delivery of virtual care, which could provide complementary insights and perspectives. Finally, the study did not assess the long-term impact of virtual care on health outcomes or health care utilization, which warrants further investigation to fully understand the effectiveness and feasibility of virtual care for this population.

### Conclusion

In conclusion, this study illuminates the perceptions of diverse older adults living with HIV concerning virtual care, emphasizing the pivotal role of trust, privacy, and technology access. Using an interpretive qualitative descriptive methodology, we gleaned nuanced insights into participants’ preferences and experiences, offering actionable implications for practice and policy. Our findings underscore the imperative of cultivating trusting relationships between health care providers and older adults living with HIV in virtual care settings, necessitating strategies to ensure patient privacy, confidentiality, and cultural competence. Moreover, equitable access to technology emerges as a crucial consideration, with efforts needed to address obstacles such as poor connectivity and technological literacy. Moving forward, collaboration between health care providers and policymakers is essential to develop inclusive virtual care models that meet the diverse needs of this population, ensuring continuity of care, providing technological support, and integrating virtual care seamlessly into existing health care services. While the study’s findings provide valuable insights, limitations such as sample scope and generalizability underscore the need for further research to comprehensively understand the long-term impact of virtual care on health outcomes and health care utilization among older adults living with HIV. By embracing the unique perspectives and experiences of this population, we can work toward building more inclusive and responsive health care systems that meet the needs of all individuals, regardless of age, HIV status, or other intersecting identities.

## Supplementary material

10.2196/65730 Multimedia Appendix 1 Supplementary tables.
