# Supplementary material for: Exploring the Perspectives of Older Adults Living With HIV on Virtual Care: Qualitative Study
Source: JMIR Aging. 2024 Dec 4;7:e65730. doi: 10.2196/65730 (PMC11633518; doi:10.2196/65730)
Supplement: Multimedia Appendix 1 [file aging-v7-e65730-s001.docx]

**Table S1: Sample interview guide**

| **Sample main question** | **Sample probe** |
| --- | --- |
| Do you have any experience with accessing virtual care for health? | Was your experience with virtual care positive or negative? What about the experience made it positive? What about the experience made it negative? |
| What is important for healthcare providers to know when we think about offering you virtual care? | What type of technology would you want to use e.g., phone, tablet, call? Zoom, Teams, Skype? Why do they prefer virtual? Why in-person? Does it depend? (What type of care is better in-person vs virtual?) With what providers etc? |
| Please describe any particular challenges you might have in accessing virtual care, |  |

**Table S2: Braun and Clarke’s 15-point checklist of criteria for good thematic analysis (Braun & Clarke, 2006)[41]**

| **Criteria** | **Research Team’s Reflection** |
| --- | --- |
| The data have been transcribed to an appropriate level of detail, and the transcripts have been checked against the tapes for ‘accuracy’. | Transcripts were transcribed in full by a professional transcriptionist. The research assistant reviewed the transcripts for accuracy. |
| Each data item has been given equal attention in the coding process. | Equal attention was given to each transcript and identified concept throughout the coding process. |
| Themes have not been generated from a few vivid examples (an anecdotal approach), but instead the coding process has been thorough, inclusive, and comprehensive. | Data analysis involved a review and re-review of data, from multiple researchers, including peer-researchers with lived experience. |
| All relevant extracts for each theme have been collated. | All relevant extracts for all each theme were collated into a mind map [64]. |
| Themes have been checked against each other and back to the original data set. | Each theme was scrutinized to confirm its consistency with the data and to verify that it accurately represented the information captured in the interviews. |
| Themes are internally coherent, consistent, and distinctive. | During the analysis process, themes were examined to ensure that they were logically connected, free from contradictions, and clearly differentiated from one another. |
| Data have been analyzed − interpreted, made sense of − rather than just paraphrased or described. | This was accomplished by moving beyond simple paraphrasing or description of the data and instead engaging in thorough. Analysis involved delving deeper into the data to uncover underlying meanings, patterns, and implications, contributing to a more comprehensive understanding of the phenomena under investigation. |
| Analysis and data match each other − the extracts illustrate the analytic claims. | This was ensured by aligning the analysis with the data, ensuring that the interpretations and claims made in the analysis were supported by relevant extracts from the dataset. Extracts from the data were carefully selected to illustrate the analytical findings, enhancing the credibility and trustworthiness of the analysis. |
| Analysis tells a convincing and well-organized story about the data and topic. | Feedback from our advisory committee suggest that the data presents a coherent and well-organized narrative that effectively conveyed insights about the data and topic at hand. Through logical sequencing and clear articulation of findings, the analysis provided a compelling and comprehensive understanding of the subject matter, enhancing the overall quality and persuasiveness of the research. |
| A good balance between analytic narrative and illustrative extracts is provided. | The analysis struck a balanced approach between providing a cohesive narrative and including illustrative extracts from the data. This ensured that key insights and interpretations were effectively supported by relevant examples and quotations, enhancing the richness and depth of the analysis while maintaining clarity and coherence in the overall presentation. |
| Enough time has been allocated to complete all phases of the analysis adequately, without rushing a phase or giving it a once-over-lightly | Data collection and analysis occurred for almost an 8-month period, allowing for sufficient time. |
| The assumptions about, and specific approach to, thematic analysis is clearly explicated | Yes, see methods. |
| There is a good fit between what you claim you do, and what you show you have done − i.e., described method and reported analysis are consistent. | The reported analysis was consistent with the described method, demonstrating a strong alignment between what was claimed to be done and what was carried out. |
| The language and concepts used in the report are consistent with the epistemological position of the analysis. | The language and concepts employed in the report are congruent with the framework adopted for the analysis. |
| The researcher is positioned as active in the research process; themes do not just ‘emerge’. | Our methods highlight the deliberate and systematic approach taken by the researcher in analyzing the data, ensuring that themes are not merely perceived as spontaneously arising, but are instead actively identified, interpreted, and developed through rigorous analysis. |

**Table S3. Additional demographic information for participants.**

| **Demographic Characteristic** | **Response Option(s)** | **Number (N) (Percentage)** |
| --- | --- | --- |
| **Living Setting**  n = 14 | Urban  Suburban  Rural | 9 (64%)  2 (14%)  3 (21%) |
| **Country of Birth**  n = 14 | Canada  Other | 6 (43%)  8 (57%) |
| **Length of time residing in Canada**  n = 14 | 0-5 years  6-10 years  More than 10 years | 4 (29%)  1 (7%)  9 (64%) |
| **Ethnicity**  n = 14  ***Some participants selected more than one ethnicity.** | Asian – East (e.g. Chinese, Japanese, Korean)  Asian – South (e.g. Indian, Pakistani, Sri Lankan)  Asian – South East (e.g. Malaysian, Filipino, Vietnamese)  Black – African (e.g. Ghanaian, Kenya, Somali)  Black – North American (e.g. Canadian, American) Indian –  Caribbean (e.g. Guyanese with origins in India)  Indigenous  Latin American (e.g. Argentinean, Chilean, Salvadoran)  Middle Eastern (e.g. Egyptian, Iranian, Lebanese)  White – European (e.g. English, Italian, Portuguese)  White – North American (e.g. Canadian, American)  Mixed heritage (e.g. Black-African & White – North American) | 0 (0%)  3 (19%)  0 (0%)  1 (6%)  0 (0%)  1 (6%)  2 (13%)  0 (0%)  0 (0%)  4 (25%)  4 (25%)  1 (6%) |
| **Sexual Orientation**  n = 14 | Heterosexual (straight, men/women relationships)  Bisexual  Gay  Lesbian  Queer  Two-Spirit  Asexual | 5 (35%)  0 (0%)  7 (50%)  0 (0%)  0 (0%)  0 (0%)  2 (14%) |
| **Household income before taxes**  n = 14 | $0 - $29,999  $30,000 - $59,999  $60,000 - $89,999  $90,000 - $119,999  $120,000 - $149,999  $150,000 or more  Don’t Know | 9 (64%)  0 (0%)  3 (21%)  1 (7%)  0 (0%)  1 (7%)  0 (0%) |
| **Number of people in household**  n = 14 | 0  1  2 | 1 (Noted Precariously Housed;7%)  8 (57%)  5 (36%) |
| **Religion or spiritual affiliation**  n = 14 | Agnosticism (Agnostic)  Atheism (Atheist)  Buddhism (Buddhist) Christianity (Christian)  Hinduism (Hindu)  Indigenous spirituality  Islam (Muslim)  Judaism (Jewish)  Sikhism (Sikh)  Spiritual  No religion  More than one faith or religion | 1 (7%)  1 (7%)  0 (0%)  7 (50%)  1 (7%)  1 (7%)  1 (7%)  0 (0%)  0 (0%)  2 (14%)  0 (0%)  0 (0%) |
| **Level of education**  n = 14 | Less than high school  High school or equivalent  Some college or university Degree or diploma from a College or University  Graduate or professional degree | 3 (21%)  2 (14%)  3 (21%)  3 (21%)  3 (21%) |
| **Disabilities**  n = 14 | A sensory impairment (vision)  A sensory impairment (hearing) A mobility impairment (e.g., use of a cane)  A learning disability (e.g., ADHD, dyslexia)  A mental health disorder  A disability or impairment not listed above  No disability or impairment  No response | 5 (36%)  1 (7%)  2 (14%)  0 (0%)  3 (21%)  0 (0%)  3 (21%)  4 (29%) |
| **Comorbidities**  n = 14 | Diabetes  High blood pressure  High cholesterol  Heart disease  Cancer  COPD  Asthma  Arthritis  Dementia  Chronic pain  Diagnosed Mental health  Other  No response | 0 (0%)  3 (21%)  3 (21%)  0 (0%)  2 (14%)  1 (7%)  1 (7%)  2 (14%)  1 (7%)  3 (21%)  3 (21%)  1 (7%)  4 (29%) |
